# Fabulous but Forgotten Fucoid Forests

**DOI:** 10.1002/ece3.70491

**Published:** 2024-11-09

**Authors:** Mads S. Thomsen, Peter A. U. Stæhr, Paul M. South

**Affiliations:** ^1^ Marine Ecology Research Group School of Biological Sciences, University of Canterbury Christchurch New Zealand; ^2^ Department of Ecoscience Aarhus University Roskilde Denmark; ^3^ Cawthron Institute Nelson New Zealand

**Keywords:** brown seaweed, ecological functions, ecosystem services, habitat‐formers, marine forests, primary and alternative foundation species, under‐valued

## Abstract

Fucoid forests are areas dominated by marine brown seaweed in the taxonomic order Fucales that, like the better‐known marine foundation species—corals, kelps, seagrasses, salt marshes, and mangroves—are threatened by anthropogenic stressors. Fucoid forests are fabulous and important because they, like the better‐known marine foundation species (i) span large areas, bioregions, and ecosystems, (ii) provide ecological functions such as high productivity, biodiversity, and habitat for iconic and endemic species, and (iii) support a variety of ecosystem services, like commercial fisheries, regulation of nutrients and carbon, and cultural values. Fucoid forests are, based on a new citation analysis, forgotten worldwide, because they are described orders of magnitude less than the better‐known marine foundation species, in ecology and marine biology textbooks, in Google Scholar and Scopus databases over scientific literature, and in recent reports and reviews about seaweed forests. Fucoid forests would be less forgotten if more people acknowledge their biological importance and societal value more often and equate their importance to that of the better‐known marine foundation species. To decrease the knowledge gap between fucoids and the better‐known foundation species, researchers and science communicators could join forces under a broad “fucoid umbrella,” establish stronger online presences, coordinate and collaborate on publications, and produce free eye‐catching non‐technical materials for teachers, managers, politicians, grass‐root organizations, philanthropists, and funding agencies.

## Graphical abstract

We describe that fucoid seaweed forests are vital ecosystems around the world, but also document that they are much less known compared to coral reefs, kelp forests, seagrass meadows, saltmarshes, and mangroves. We hope our short easy read viewpoint will make fucoid forest more memorable, more valued, and better conserved and managed.

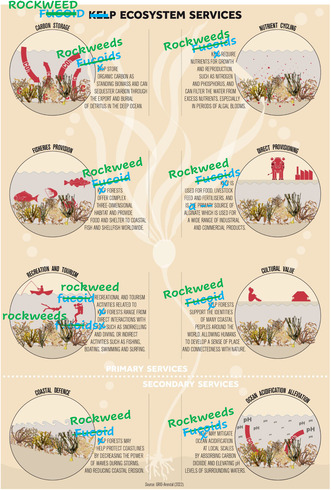

## 1 Introduction: A Forgotten Paper About a Forgotten Topic?

In 2017, an important review was published in Ecology & Evolution, with the objective of highlighting that underwater forests dominated by fucoid seaweeds are vital ecosystems, but have been “forgotten” (Coleman and Wernberg 2017). Today, this paper has been cited 73 times (Scopus search, 31/1‐2024), but only by marine biologists and only in research papers that focused exclusively on marine ecosystems. This suggests that take‐home messages about the broad importance of fucoid forests have not expanded into general branches of ecology and conservation, perhaps because the paper focused on detailed fucoid biology in a regional context (the subtidal reefs of temperate Australia).

In this viewpoint, we aim to restate and broaden the essential take home message of Coleman and Wernberg (2017) to *all* readers of Ecology & Evolution: Fucoid forests are globally important ecosystems and should not be forgotten!

## 2 What Are Fucoid Forests?

Fucoids refers to species of seaweeds that belong to the order Fucales (Phaeophycae, i.e., brown macroalgae), a monophyletic taxonomic group represented by over 550 species from 51 genera and 9 families (Algabase 2 Dec 2023; Bringloe et al. 2020). Most fucoids are large species (by marine standards) that separated over 60 million years ago from the other brown seaweeds (Bringloe et al. 2020). By comparison, the better‐known kelps are large brown seaweed in the order Laminariales (Schiel and Foster 2006; Coleman and Wernberg 2017), although some seaweed researchers emphasize ecological function over taxonomy and therefore argue that the term “kelps” should also include large non‐laminarian brown seaweed (Fraser 2012; Wernberg and Filbee‐Dexter 2019). Terrestrial forests are composed of trees (large wooden angiosperms) that are foundational species of great ecological importance (Ellison et al. 2005; Ellison 2019). In marine biology, areas dominated by relatively large ecologically important foundational species, such as seagrasses (marine angiosperms) and seaweed (red, green, and brown marine macroalga) are generally referred to as marine forests. Furthermore, in marine biology, forests are typically interpreted in a seascape context, where the sizes of the foundation species are viewed from the perspective of the animals that live in, on, and around them (Boström et al. 2011; Tokeshi and Arakaki 2012; Wernberg and Filbee‐Dexter 2019). Therefore, seagrasses and seaweeds that are smaller than terrestrial trees are still functionally “trees” (e.g., most marine forest in Australia and Europe are only a few meters tall; Smale et al. 2013; Bennett et al. 2015; Coleman and Wernberg 2017). Thus, although some researchers split areas dominated by large, intermediate, and small seaweeds, into forests, beds/meadows, and lawns/turfs (Connell, Foster, and Airoldi 2014; Wernberg and Filbee‐Dexter 2019), respectively, areas inhabited by dense stands of fucoids still function as “forests” for the many species living in them.

In short, areas dominated by fucoid seaweeds are fucoid forests.

## 3 Why Are Fucoid Forests Important?

**FIGURE 1:**
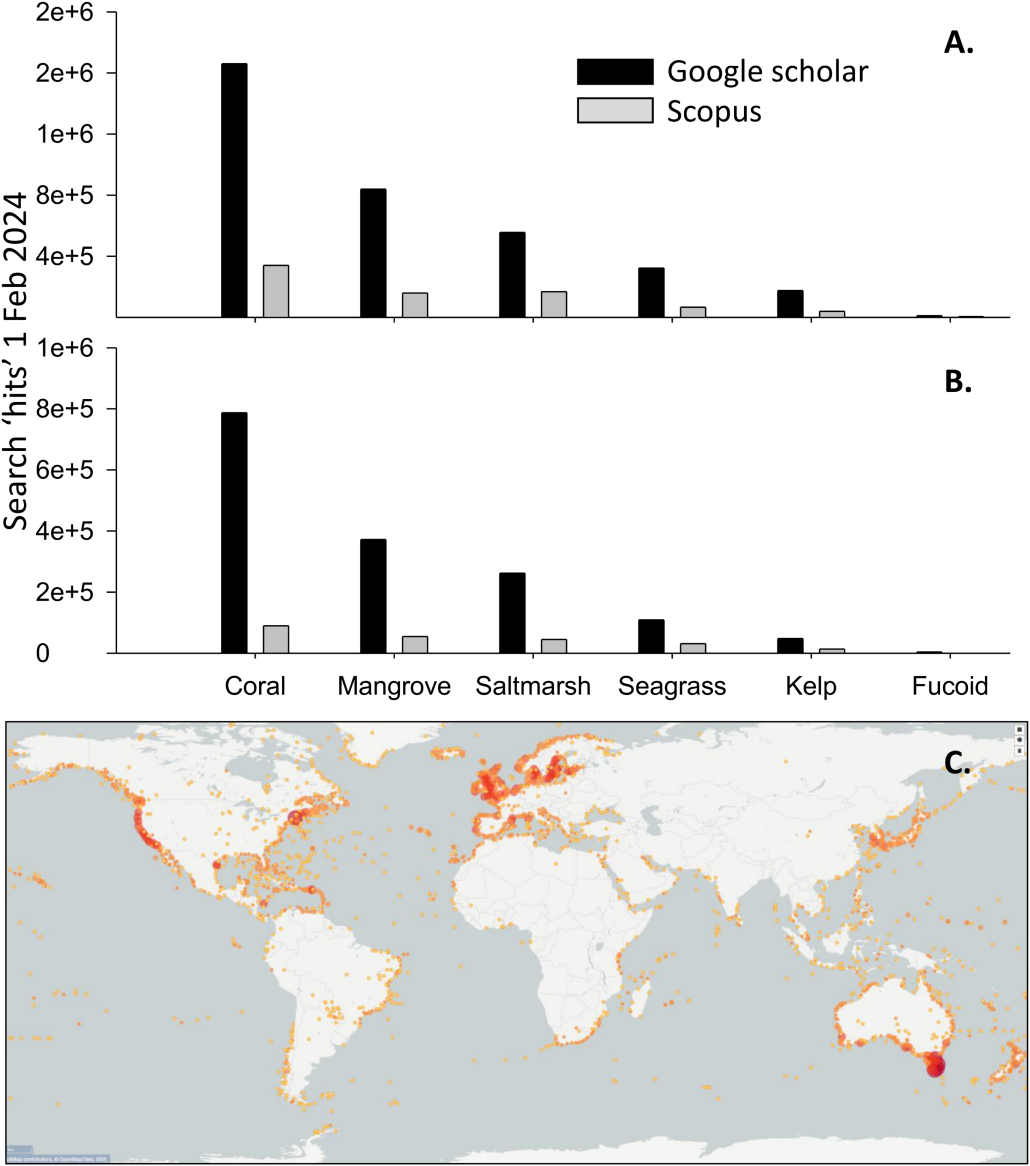
Fucoid literature compared to other marine foundation species and the global distribution of fucoids. (A) Search engine hits (1 Feb 2024) comparing fucoids to other coastal foundational taxa ‘alone’ (A) and with “AND conservation” (B). Global distribution of fucoids in the GBIF database (C, 637,617 georeferenced records, February 28, 2024). Land records may represent locations of stored specimens whereas coastal absences partly reflect lack of reporting (e.g., fucoids do occur along coastlines in Namibia, Angola, northern Chile, and southern Peru). Open water observations could be rafting of buoyant drift aggregations like *Sargassaum fluitans/natans* in the Atlantic or *Durvillaea antarctica* in the Southern Ocean. True absences may occur around Antarctica and perhaps the most northern Russia, Greenland and Canada.

**FIGURE 2:**
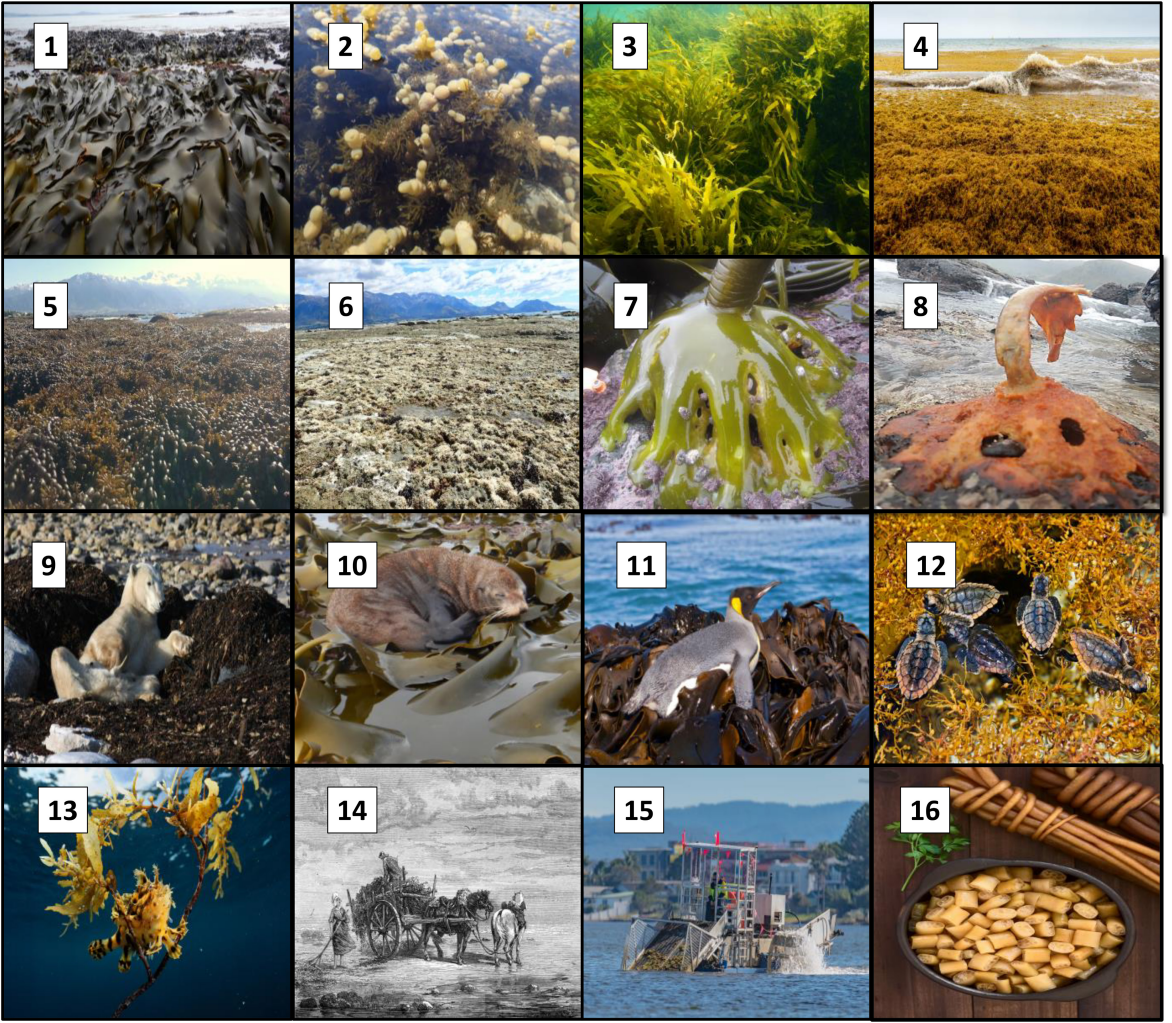
Collage depicting ecological and socioeconomic importance of fucoids. 1. Intertidal forests of the world's largest fucoid (> 10 m *Durvillaea*). 2. The world's smallest fucoid (< 5 cm *Notheia anomala*) as an obligate epiphyte on its fucoid host, *Hormosira banksii*. 3. Subtidal fucoid forests of reintroduced and restored *Phyllospora comosa* (‘Operation Crayweed’, Vergés et al. 2020), 4. Fucoid beach wrack, arising from the world's largest seaweed forest of pelagic *Sargassum* spp., 5. Diverse fucoid forests in Kaikoura with 12 cooccurring fucoid species (*Hormosira banksii*, *Notheia anomala*, *Landsburgia quercifolia, Sargassum sinclarii, 2 Marginariella species*, 2 *Carpophyllum* species, 2 *Durvillaea* species, and 3 *Cystophora* species). 6. Same location as in ‘5’ showing destroyed fucoid forests after a natural disaster (seismic uplift in November 2016). 7. Fucoid holdfasts inhabited by > 50 invertebrate species (*Durvillaea*). 8. Same location as in ‘7’ showing a dying fucoid ‘ghost’ holdfast leading to regional extinction after the 2017/18 Tasman Sea heatwave. 9–13 Examples of fucoid ‘beds’ (literally) for charismatic animals—a polar bear resting on arctic *Fucus* wrack, a fur seal and snares penguins resting on intertidal *Durvillaea* in New Zealand and its subantarctic islands, and juvenile Loggerhead turtles and co‐evolved camouflaged *Sargassum* frog fish resting on, feeding within, and avoiding predators in floating pelagic *Sargassum* forests. 14–15. Fucoids as socioeconomic resources showing historical and present‐day harvest of beach wrack and living fucoid forests. 16. Fucoid (cochayuyo/*Durvillaea antarctica*) as a dish eaten today and 14,000 years ago by the first settlers of South America, fossilized in the Verde‐caves and has perpetuated common vernacular with ‘remojar el cochayuyo’ (i.e., ‘soaking the cochayuyo’) being slang for sexual intercourse. Photo 1–3, 5–9, 10 by M. Thomsen, 4, 9, 11–16 are alamy Stock Photo Inc. www.alamy.com (ID numbers; 2J0618Y, BK6YHM, C02031, A04M5Y, W85DCC, FG6YE7, 2CB2CCN, and M4Y7XT, respectively).

**FIGURE 3:**
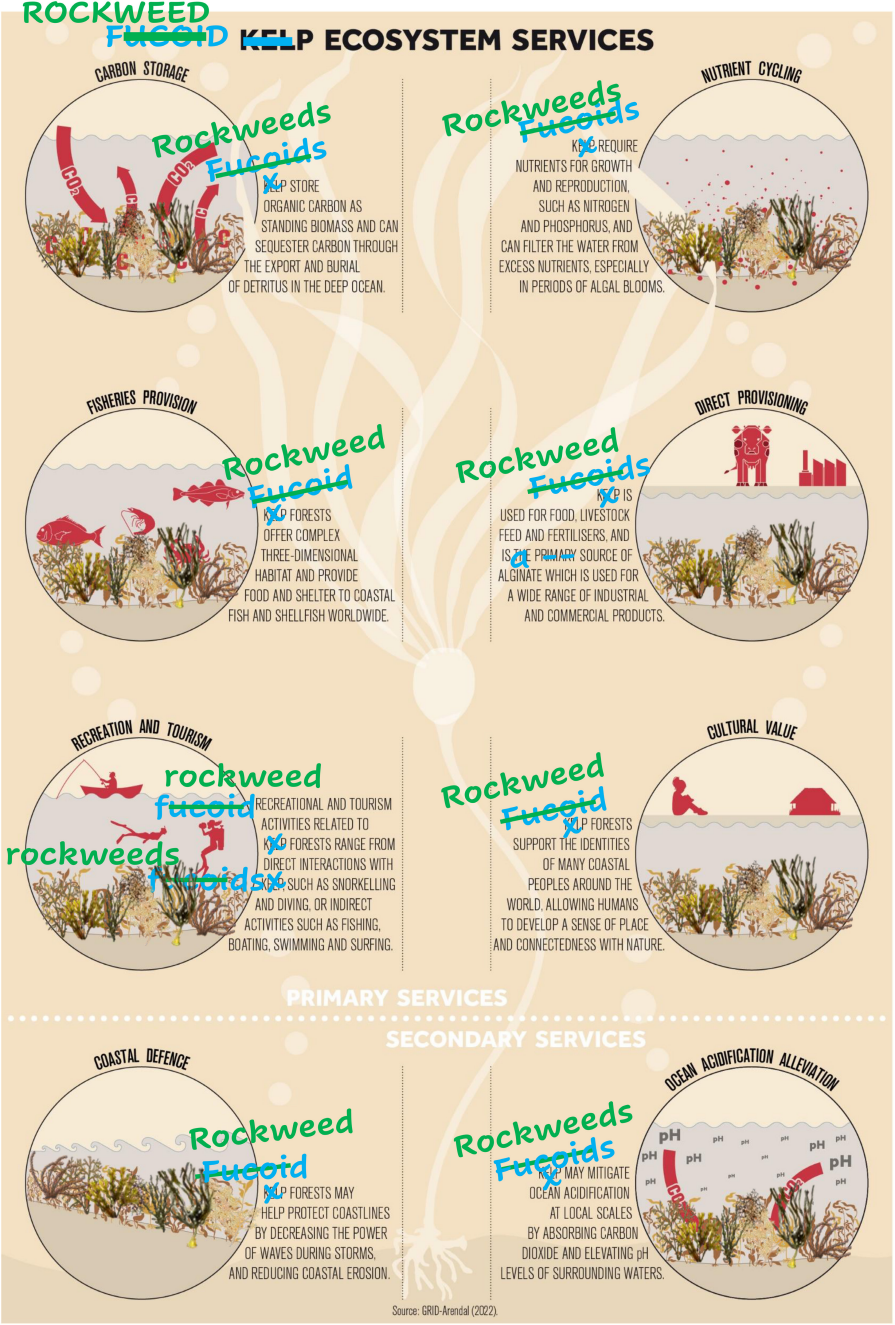
Ecosystem services in rockweed forests (modified slightly from Figure 3.2 in Smale et al. 2023—source GRID‐Arendal 2022). Disregarding that some researchers and dictionaries call fucoids for “rockweeds,” others classify them as a subgroup of kelps, and most only name them fucoids (see Section 5 for details), we see many opportunities to increase knowledge and appreciation of their value, e.g., by highlighting their commonality and numerous ecosystem services.

First, fucoids cover vast areas, estimated to 2.57 million km^2^ (Fragkopoulou et al. 2022). This area is larger than the estimated global coverages of other marine foundational species, including the true laminarian kelps (c. 1.70 million km^2^), seagrasses (c. 1.65 million km^2^), coral reefs (c. 0.28 million km^2^), mangroves (c. 0.15 million km^2^), and salt marshes (c. 0.06 million km^2^; Jayathilake and Costello 2018; Davidson et al. 2019; McKenzie et al. 2020; Bunting et al. 2022; Fragkopoulou et al. 2022). Indeed, fucoids are more widely distributed compared to many other marine foundation species because they are found worldwide in all biogeographical realms (Figure 1), creating dense forests along large stretches of coastlines, such as in the Baltic sea (Torn, Krause‐Jensen, and Martin 2006), Mediterranean sea (Ballesteros et al. 2009; Rendina et al. 2023), Australia (Coleman and Wernberg 2017), New Zealand (Schiel 1990), many tropical coastlines (Fragkopoulou et al. 2022), and even in the open ocean (Brooks et al. 2018; Wang et al. 2019). For example, in 2018, more than 20 million tons of floating *Sargassum natans* and *S. fluitans* covered c. 6000 km^2^ in the tropical part of the Atlantic ocean (Figure 2), forming one of the largest near‐monospecific marine habitats on the planet (Wang et al. 2019). Second, fucoids have evolved diverse eco‐physiological traits allowing them to create forests under widely different environmental conditions, including (a) from cold polar (e.g., *Ascophyllum*) to hot tropical (e.g., *Turbinaria*) latitudes, (b) in waves exposed (e.g., *Durvillaea*) and sheltered (e.g., *Hormosira*) locations, (c) from desiccation stressed intertidal (e.g., *Pelvetia*), to light stressed deep waters (e.g., *Cystoseira*), (e) on bottom substrates (most fucoids) and the open pelagic (*Sargassum*), and (f) in both brackish waters like the Baltic sea and estuaries (e.g., *Fucus*) and fully marine oceans (all fucoids; King 1981; Torn, Krause‐Jensen, and Martin 2006; Ballesteros et al. 2009; Thomsen and South 2019; Wang et al. 2019; Fragkopoulou et al. 2022; Pessarrodona and Grimaldi 2022). Furthermore, the ability of some fucoids to grow vegetatively allows population to dominate as drift fucoids in sheltered muddy systems, including marshes, mudflats, and mangroves (e.g., *Fucus, Hormosira*), and even as epiphytes attached to other fucoids species (*Notheia*; e.g., Schiel and Foster 2006; Tuya and Haroun 2006; Thomsen et al. 2015; Coleman and Wernberg 2017; Martínez et al. 2018; Barboza et al. 2019; Cheung‐Wong et al. 2022). Indeed, form‐functional trait values that typically increase a species' ecological importance, are often higher for fucoids compared to similar trait values associated with other marine foundation species. For example, fucoids can be very large and heavy (70 kg and > 10 m fronds for *Durvillaea* species) and long‐lived (> 50 years for *Ascophyllum*) and can hold and dominate space on long time scales (Åberg 1992; Hurd 2003; Menge et al. 2017; Vaux et al. 2023). Third, fucoids provide important ecological functions and ecosystem services. Common ecological functions include high productivity, supporting complex food webs and biodiversity, modifying environmental conditions ‐ and many fucoids are consequently considered indicator species for ecosystem health (Schiel and Foster 2006; Kersen et al. 2011; Schagerström et al. 2014; Coleman and Wernberg 2017; Vasconcelos and de Vasconcelos Reis 2019; Benes and Bracken 2020; Pessarrodona et al. 2022; Thomsen et al. 2022). For example, fucoids, like other marine foundation species, build complex biogenic habitat, represented by many different morphological shapes and sizes of their holdfast, stipes, and fronds (e.g., with bladed or branched morphologies and sometimes air bladders and unique reproductive structures; Mattern et al. 2009; Figure 2). These biogenic structures provide habitat and nursery and feeding grounds for many iconic and endemic species, including *Chelonia mydas* (green turtle), *Cephalorhynchus hectori* (Hector's dolphin), *Arctocephalus forsteri* (New Zealand fur seal), *Eudyptes robustus* (Snares penguin), *Histrio histrio* (sargassum frog fish), *Macroctopus maorum* (Māori octopus), and *Hippocampus abdominalis* (big‐belly seahorse; Warham 1974; Grubert, Wadley, and White 1999; Witherington, Hirama, and Hardy 2012; Miller 2015; Bartes et al. 2024). Fucoids also provide essential ecosystem services, such as nutrient retention, carbon sequestration, habitats for commercial fishery species, food for humans, and a variety of cultural benefits (Figure 3, Ugarte and Sharp 2001; Bellgrove et al. 2017; Coleman and Wernberg 2017; De La Fuente et al. 2019; Vergés et al. 2020; Duarte et al. 2022; Fragkopoulou et al. 2022).

Finally, fucoids are threatened by the same suite of anthropogenic stressors that are impacting other marine foundation species around the world, including stronger heatwaves, sediment‐smothering, overgrazing, biological invasions, coastal darkening, and eutrophication (Kautsky et al. 1986; Smale and Wernberg 2013; Valdazo et al. 2017; Schiel et al. 2019; Valdazo, Viera‐Rodríguez, and Tuya 2020; Thomsen et al. 2021; Verdura et al. 2021; Fragkopoulou et al. 2022; Pessarrodona 2022; Wernberg et al. 2024). Fucoids should therefore be included in conservation and restoration to the same extent as other marine foundation species (Bellgrove et al. 2010, 2017; Vergés et al. 2020; Eger et al. 2022b; Whitaker et al. 2023).

In short, fucoids are important because they, like other marine foundation species, cover vast areas, bioregions, and ecosystems, provide vital ecological functions and ecosystem services and are also threatened by human activities.

## 4 Are Fucoid Forests Forgotten?

Coleman and Wernberg (2017) argued that subtidal fucoid forests in temperate Australia are forgotten because they identified five times more research papers done on laminarian kelps. Here we list more examples that suggest fucoid forests are forgotten beyond subtidal forests in temperate Australia. First, our citation analysis of Coleman and Wernberg (2017) suggested that this important review, did not, when presented to the broad readership of Ecology and Evolution, move into the non‐marine literature (see Section 1). Perhaps, this is not surprising because other citation analyses suggest that marine research is cited less frequently by terrestrial researchers than vice versa (Menge et al. 2009a, 2009b). Second, a new keyword search in 10 biology and ecology textbooks taught in many university courses, showed that fucoids are mentioned orders of magnitude less than other marine foundation species. We found that across the 10 textbooks, “fucoid” was only referenced 4 times whereas there were 193 references to “kelp,” 1035 to “coral,” 45 to “seagrass,” 198 to “mangrove,” and 328 to “salt marsh” (including “saltmarsh”; Verhoef and Morin 2010; Krebs 2014; Smith and Smith 2015; Campbell et al. 2017; Fowler, Roush, and Wise 2017; Mittelbach and McGill 2019; Molles Jr and Sher 2019; Clark, Choi, and Douglas 2020; Begon and Townsend 2021; Keddy and Laughlin 2021). Similar discrepancies were found in five marine textbooks used to teach future marine biologists, managers and conservationists, with only 5 references to fucoid compared to 425 to kelp, 2465 to coral, 914 to seagrass, 759 to mangrove, and 411 to salt marsh/saltmarsh (Lalli and Parsons 1997; Kaiser et al. 2011; Ray and McCormick‐Ray 2013; Castro and Huber 2019; Duffy 2021). Third, wider searches were done in Scopus and Google Scholar (February 1, 2024) to compare the total available information in reports, theses, and research papers between fucoids and the better known‐marine foundation species. We used the same single word searches for each marine foundation species, but also searched for available information related to management, by combining individual marine foundation species with the keywords “AND conservation”. Across all keywords and search engines there was orders of magnitude less information available for fucoids compared to other marine foundation species, with 79–194× more hits for corals, 15–29× more for seagrasses, 37–92× more for mangroves, 39–65× more for saltmarshes/salt marshes, and 9–16× more for kelps (Figure 1A,B). Finally, there have been recent, targeted efforts to assign greater value to seaweed forests, resulting in several global reports and review papers about their biology, ecology, threats, services, management, restoration, and values to humans (e.g., Eger et al. 2022a, 2022b, 2023; Eger 2023; Lutz 2023). These outputs have focused almost exclusively on kelps and kelp terminology (3710 references to kelp vs. 10 to fucoids, although some of these publications and in contrast to most of the scientific literature, classified fucoids as a subgroup of kelp, again resulting in fewer citations and reduced recognition).

In short, fucoids are forgotten because they are described orders of magnitude less than similar important marine foundation species in biology, ecology, and marine textbooks, in online search engines and in report and reviews about the broad importance of brown seaweed forests.

## 5 What Can Be Done to Better Know, Teach, Study, Value, and Conserve Forgotten Fucoid Forests?

Fucoid forests are simple to describe (Section 2), are important (Section 3), but forgotten (Section 4). However, fucoid forests would become better known if more people, outside the world of seaweed experts, acknowledge their biological importance and societal value more often. The aim of our viewpoint is to make more people aware of fucoids and their importance, like they are about kelps, corals, seagrasses, mangroves, seagrasses, and salt marshes. Increased knowledge and appreciation of fucoid forests could then spill over into broader teaching, research, management, conservation and a better understanding of ecosystem interconnectedness (Carr et al. 2003; Webb 2012). Interestingly, the fucoid name mirrors the Latin order “Fucales,” the family “Fucacea” and the genus “Fucus,” contrasting the non‐Latin common names for the better‐known corals, kelps, seagrasses, salt marshes, and mangroves. Perhaps, that is why some researchers have called fucoids “rockweeds” (Estes, Duggins, and Rathbun 1989; Steinberg, Estes, and Winter 1995; Van Alstyne et al. 1999a, 1999b; Tegner and Dayton 2000; Liu et al. 2012; Agatsuma 2014; Duffy 2021; Elsberry and Bracken 2021), similar to Webster's Dictionary and The Free Dictionary (“rockweeds are any of various coarse brown algae in the order Fucales” Anon 2021a, 2021b). However, rockweeds have more traditionally referred only to northern hemisphere species in the Fucaceae family (Chapman 1989; McCook and Chapman 1993; Murray and Denis 1997; Denis 2003; Larsen 2012). Irrespective of diverging opinions about whether fucoids should be called rockweeds (e.g., Estes, Duggins, and Rathbun 1989; Steinberg, Estes, and Winter 1995; Van Alstyne et al. 1999b; Tegner and Dayton 2000; Anon. 2021b; Duffy 2021), should be classified as kelps (Eger et al. 2022a, Eger et al. 2022b) or should continue to be called fucoids (the majority of research), we see many opportunities to increase knowledge and appreciation of marine forests where these brown seaweed are common or dominate. First, researchers and managers could form international working groups and networks, organize meetings to learn from one another, arrange international data collections and experiments, and try to publish results in high impact globally oriented journals—as well as encourage the United Nations and other international agencies to commission reports about fucoids. Second, researchers and managers could, with the help of international, national, and regional agencies and citizen science projects, build online databases containing standardized data about the distribution and abundances of fucoids and their co‐occurring species. Third, researchers and managers could allocate more time to engage science communicators to help produce visually appealing free materials aimed at teachers, managers, politicians, grassroots organizations, philanthropists, and funding agencies. Finally, researchers and managers could aim for a more conspicuous online presence, such as by expanding the Wikipedia pages for fucoids and Fucales (less than one page with virtually no information as per 21/8/2024), building and maintaining more webpages that focus on fucoids, and by creating fucoid‐related content and communications on social media to highlight fucoid biology, ecology, and importance for humans.

In short, we hope fucoids will become better known and valued more and to achieve this objective, more scientists, managers, and conservationists, should elevate fucoids to the level of better‐known marine foundation species more often, irrespective of their study field.

## Author Contributions

**Mads S. Thomsen:** conceptualization (equal), writing – original draft (lead). **Paul M. South:** conceptualization (equal), writing – review and editing (equal). **Peter A. U. Stæhr:** conceptualization (equal), writing – review and editing (equal).

## Conflicts of Interest

The authors declare no conflicts of interest.

## Data Availability

The authors have nothing to report.

## References

[ece370491-bib-0001] Åberg, P. 1992. “A Demographic Study of Two Populations of the Seaweed *Ascophyllum nodosum* .” Ecology 73: 1473–1487.

[ece370491-bib-0002] Agatsuma, Y. 2014. “Population Dynamics of Edible Sea Urchins Associated With Variability of Seaweed Beds in Northern Japan.” Auqa‐BioScience Monographs 7: 47–78.

[ece370491-bib-0003] Anon . 2021a. “Rockweeds: Any of Various Brown Algae of the Order Fucales Growing Chiefly on Rocks in Coastal Areas, Such as Bladder Wrack or *Ascophyllum nodosum*.” https://www.thefreedictionary.com/rockweed.

[ece370491-bib-0004] Anon . 2021b. “Rockweeds: Any of Various Brown Algae of the Order FUCALES Growing Chiefly on Rocks in Coastal Areas, Such as Bladder Wrack or *Ascophyllum nodosum*.” https://www.merriam‐webster.com/dictionary/rockweed.

[ece370491-bib-0005] Ballesteros, E. , J. Garrabou , B. Hereu , M. Zabala , E. Cebrian , and E. Sala . 2009. “Deep‐Water Stands of *Cystoseira zosteroides* C. Agardh (Fucales, Ochrophyta) in the Northwestern Mediterranean: Insights Into Assemblage Structure and Population Dynamics.” Estuarine, Coastal and Shelf Science 82: 477–484.

[ece370491-bib-0006] Barboza, F. R. , J. Kotta , F. Weinberger , et al. 2019. “Geographic Variation in Fitness‐Related Traits of the Bladderwrack *Fucus vesiculosus* Along the Baltic Sea‐North Sea Salinity Gradient.” Ecology and Evolution 9: 9225–9238. 31463018 10.1002/ece3.5470 PMC6706220

[ece370491-bib-0007] Bartes, S. N. , J. Monk , C. Jenkins , M. A. Hindell , D. P. Costa , and J. P. Y. Arnould . 2024. “Habitat Selection and Influence on Foraging Success in Female Australian Fur Seals.” 10.1038/s41598-024-78643-5 PMC11541878 39506103

[ece370491-bib-0008] Begon, M. , and C. R. Townsend . 2021. Ecology—From Individuals to Ecosystems. 5th ed. Massachusetts: Blackwell Publishing.

[ece370491-bib-0009] Bellgrove, A. , P. F. McKenzie , H. Cameron , and J. B. Pocklington . 2017. “Restoring Rocky Intertidal Communities: Lessons From a Benthic Macroalgal Ecosystem Engineer.” Marine Pollution Bulletin 117: 17–27. 28202275 10.1016/j.marpolbul.2017.02.012

[ece370491-bib-0010] Bellgrove, A. , P. F. McKenzie , J. L. McKenzie , and B. J. Sfiligoj . 2010. “Restoration of the Habitat‐Forming Fucoid Alga *Hormosira banksii* at Effluent‐Affected Sites: Competitive Exclusion by Coralline Turfs.” Marine Ecology Progress Series 419: 47–56.

[ece370491-bib-0011] Benes, K. , and M. E. S. Bracken . 2020. “Interactive Effects of Large‐and Local‐Scale Environmental Gradients on Phenotypic Differentiation.” Ecology 101: e03078. 32542682 10.1002/ecy.3078

[ece370491-bib-0012] Bennett, S. , T. Wernberg , S. D. Connell , A. J. Hobday , C. R. Johnson , and E. S. Poloczanska . 2015. “The ‘Great Southern Reef’: Social, Ecological and Economic Value of Australia's Neglected Kelp Forests.” Marine and Freshwater Research 67: 47–56.

[ece370491-bib-0013] Boström, C. , S. J. Pittman , C. Simenstad , and R. T. Kneib . 2011. “Seascape Ecology of Coastal Biogenic Habitats: Advances, Gaps, and Challenges.” Marine Ecology Progress Series 427: 191–217.

[ece370491-bib-0014] Bringloe, T. T. , S. Starko , R. M. Wade , et al. 2020. “Phylogeny and Evolution of the Brown Algae.” Critical Reviews in Plant Sciences 39: 281–321.

[ece370491-bib-0015] Brooks, M. T. , V. J. Coles , R. R. Hood , and J. F. R. Gower . 2018. “Factors Controlling the Seasonal Distribution of Pelagic *Sargassum* .” Marine Ecology Progress Series 599: 1–18.

[ece370491-bib-0016] Bunting, P. , A. Rosenqvist , L. Hilarides , R. M. Lucas , and N. Thomas . 2022. “Global Mangrove Watch: Updated 2010 Mangrove Forest Extent (v2. 5).” Remote Sensing 14: 1034.

[ece370491-bib-0017] Campbell, N. A. , J. B. Reece , L. A. Urry , et al. 2017. Biology: A Global Approach. 1st ed. Harlow: Global Edition.

[ece370491-bib-0018] Carr, M. H. , J. E. Neigel , J. A. Estes , S. Andelman , R. R. Warner , and J. L. Largier . 2003. “Comparing Marine and Terrestrial Ecosystems: Implications for the Design of Coastal Marine Reserves.” Ecological Applications 13: 90–107.

[ece370491-bib-0019] Castro, P. , and M. E. Huber . 2019. Marine Biology. 11th ed. New York, NY: McGraw‐Hill Education.

[ece370491-bib-0020] Chapman, A. R. O. 1989. “Abundance of *Fucus spiralis* and Ephemeral Seaweeds in a High Eulittoral Zone: Effects of Grazers, Canopy and Substratum Type.” Marine Biology 102: 565–572.

[ece370491-bib-0021] Cheung‐Wong, R. W. Y. , J. Kotta , D. A. Hemraj , and B. D. Russell . 2022. “Persistence in a Tropical Transition Zone? *Sargassum* Forests Alternate Seasonal Growth Forms to Maintain Productivity in Warming Waters at the Expense of Annual Biomass Production.” Science of the Total Environment 851: 158154. 35995150 10.1016/j.scitotenv.2022.158154

[ece370491-bib-0022] Clark, M. A. , J. Choi , and M. Douglas . 2020. Biology, (OpenStax). Houston, Texas: OpenStax, Rice University.

[ece370491-bib-0023] Coleman, M. A. , and T. Wernberg . 2017. “Forgotten Underwater Forests: The Key Role of Fucoids on Australian Temperate Reefs.” Ecology and Evolution 7: 8406–8418. 29075458 10.1002/ece3.3279 PMC5648665

[ece370491-bib-0024] Connell, S. , M. Foster , and L. Airoldi . 2014. “What Are Algal Turfs? Towards a Better Description of Turfs.” Marine Ecology Progress Series 495: 299–307.

[ece370491-bib-0025] Davidson, N. C. , A. A. Van Dam , C. M. Finlayson , and R. J. McInnes . 2019. “Worth of Wetlands: Revised Global Monetary Values of Coastal and Inland Wetland Ecosystem Services.” Marine and Freshwater Research 70: 1189–1194.

[ece370491-bib-0026] De La Fuente, G. , M. Chiantore , V. Asnaghi , S. Kaleb , and A. Falace . 2019. “First Ex Situ Outplanting of the Habitat‐Forming Seaweed *Cystoseira amentacea* var. *stricta* From a Restoration Perspective.” PeerJ 7: e7290. 31367482 10.7717/peerj.7290 PMC6657741

[ece370491-bib-0027] Denis, T. G. 2003. “Effects of Human Foot Traffic on the Standing Stock, Reproduction, and Size Structure of Southern California Populations of the Intertidal Rockweed Silvetia Compressa (O. Fucales).” Master's thesis. California State University, Fullerton:132.

[ece370491-bib-0028] Duarte, C. M. , J. P. Gattuso , K. Hancke , et al. 2022. “Global Estimates of the Extent and Production of Macroalgal Forests.” Global Ecology and Biogeography 31: 1422–1439.

[ece370491-bib-0029] Duffy, J. E. 2021. Ocean Ecology: Marine Life in the Age of Humans. Princeton, New Jersey: Princeton University Press.

[ece370491-bib-0030] Eger, A. M. , C. Layton , T. A. McHugh , M. Gleason , and N. Eddy . 2022a. Kelp Restoration Guidebook: Lessons Learned From Kelp Restoration Projects Around the World. Vol. 85. Arlington, VA: Nature Conservancy.

[ece370491-bib-0031] Eger, A. M. , E. M. Marzinelli , R. Beas‐Luna , et al. 2023. “The Value of Ecosystem Services in Global Marine Kelp Forests.” Nature Communications 14: 1894. 10.1038/s41467-023-37385-0 PMC10113392 37072389

[ece370491-bib-0032] Eger, A. M. , E. M. Marzinelli , H. Christie , et al. 2022b. “Global kelp forest restoration: past lessons, present status, and future directions.” Biological Reviews 97: 1449–1475. 35255531 10.1111/brv.12850 PMC9543053

[ece370491-bib-0033] Eger, E. A. 2023. A Roadmap for Protecting and Restoring 4 Million Hectares of Kelp Forests by 2040, 28. Sydney, Australia: Kelp Forest Alliance.

[ece370491-bib-0034] Ellison, A. M. 2019. “Foundation Species, Non‐trophic Interactions, and the Value of Being Common.” Iscience 13: 254–268. 30870783 10.1016/j.isci.2019.02.020 PMC6416672

[ece370491-bib-0035] Ellison, A. M. , M. S. Bank , B. D. Clinton , et al. 2005. “Loss of Foundation Species: Consequences for the Structure and Dynamics of Forested Ecosystems.” Frontiers in Ecology and the Environment 3: 479–486.

[ece370491-bib-0036] Elsberry, L. A. , and M. E. Bracken . 2021. “Functional Redundancy Buffers Mobile Invertebrates Against the Loss of Foundation Species on Rocky Shores.” Marine Ecology Progress Series 673: 43–54.

[ece370491-bib-0037] Estes, J. A. , D. O. Duggins , and G. B. Rathbun . 1989. “The Ecology of Extinctions in Kelp Forest Communities.” Conservation Biology 3: 252–264.

[ece370491-bib-0038] Fowler, S. , R. Roush , and J. Wise . 2017. Concepts of Biology (OpenStax). Houston, Texas: OpenStax, Rice University.

[ece370491-bib-0039] Fragkopoulou, E. , E. A. Serrão , O. De Clerck , et al. 2022. “Global Biodiversity Patterns of Marine Forests of Brown Macroalgae.” Global Ecology Biogeography 31: 636–648.

[ece370491-bib-0040] Fraser, C. 2012. “Is Bull‐Kelp Kelp? The Role of Common Names in Science.” New Zealand Journal of Marine and Freshwater Research 46: 279–284.

[ece370491-bib-0041] Grubert, M. A. , V. A. Wadley , and R. W. G. White . 1999. “Diet and Feeding Strategy of *Octopus maorum* in Southeast Tasmania.” Bulletin of Marine Science 65: 441–451.

[ece370491-bib-0042] Hurd, C. L. 2003. Bull Kelp. In 'The Living Reef: The Ecology of New Zealand's Rocky Reefs, edited by N. Andrew and M. Francis . Nelson, New Zealand: Craig Potton Publishing.

[ece370491-bib-0043] Jayathilake, D. R. M. , and M. J. Costello . 2018. “A Modelled Global Distribution of the Seagrass Biome.” Biological Conservation 226: 120–126.

[ece370491-bib-0044] Kaiser, M. J. , S. Jennings , D. N. Thomas , and D. K. A. Barnes . 2011. Marine Ecology: Processes, Systems, and Impacts. New York: Oxford University Press.

[ece370491-bib-0045] Kautsky, N. , H. Kautsky , U. Kautsky , and M. Waern . 1986. “Decreased Depth Penetration of *Fucus vesiculosus* (L.) Since the 1940's Indicates Eutrophication of the Baltic Sea.” Marine Ecology Progress Series 28: 1–8.

[ece370491-bib-0046] Keddy, P. A. , and D. C. Laughlin . 2021. A Framework for Community Ecology: Species Pools, Filters and Traits. Cambridge, UK: University Printing House.

[ece370491-bib-0047] Kersen, P. , J. Kotta , M. Bučas , N. Kolesova , and Z. Deķere . 2011. “Epiphytes and Associated Fauna on the Brown Alga *Fucus vesiculosus* in the Baltic and the North Seas in Relation to Different Abiotic and Biotic Variables.” Marine Ecology 32: 87–95.

[ece370491-bib-0048] King, R. J. 1981. “The Free‐Living Hormosira banksii (Turner) Decaisne Associated With Mangroves in Temperate Eastern Australia.” Botanica Marina 24: 1981. 10.1515/botm.1981.24.11.569.

[ece370491-bib-0049] Krebs, C. J. 2014. Ecology; the Experimental Analysis of Distribution and Abundance, 6th Edition. Harlow, Essex: Pearson Education Limited.

[ece370491-bib-0050] Lalli, C. , and T. R. Parsons . 1997. “Introduction.” In Biological Oceanography: An Introduction. London: Elsevier.

[ece370491-bib-0051] Larsen, P. F. 2012. “The Macroinvertebrate Fauna of Rockweed (*Ascophyllum nodosum*)‐dominated Low‐Energy Rocky Shores of the Northern Gulf of Maine.” Journal of Coastal Research 28: 36–42.

[ece370491-bib-0052] Liu, L. , M. Heinrich , S. Myers , and S. A. Dworjanyn . 2012. “Towards a Better Understanding of Medicinal Uses of the Brown Seaweed *Sargassum* in Traditional Chinese Medicine: A Phytochemical and Pharmacological Review.” Journal of Ethnopharmacology 142: 591–619. 22683660 10.1016/j.jep.2012.05.046

[ece370491-bib-0053] Lutz, S. 2023. “Into the Blue: Securing a Sustainable Future for Kelp Forests.” UN Report, Grid‐Arendal, Nairobi:149.

[ece370491-bib-0054] Martínez, B. , B. Radford , M. S. Thomsen , et al. 2018. “Distribution Models Predict Large Contractions of Habitat‐Forming Seaweeds in Response to Ocean Warming.” Diversity and Distributions 24: 1350–1366.

[ece370491-bib-0055] Mattern, T. , D. M. Houston , C. Lalas , A. N. Setiawan , and L. S. Davis . 2009. “Diet Composition, Continuity in Prey Availability and Marine Habitat—Keystones to Population Stability in the Snares Penguin (*Eudyptes robustus*).” Emu—Austral Ornithology 109: 204–213.

[ece370491-bib-0056] McCook, L. J. , and A. R. O. Chapman . 1993. “Community Succession Following Massive Ice‐Scour on a Rocky Intertidal Shore: Recruitment, Competition and Predation During Early, Primary Succession.” Marine Biology 115: 565–575.

[ece370491-bib-0057] McKenzie, L. J. , L. M. Nordlund , B. L. Jones , L. C. Cullen‐Unsworth , C. Roelfsema , and R. K. F. Unsworth . 2020. “The Global Distribution of Seagrass Meadows.” Environmental Research Letters 15: 074041.

[ece370491-bib-0058] Menge, B. A. , M. E. S. Bracken , J. Lubchenco , and H. M. Leslie . 2017. “Alternative State? Experimentally Induced F Ucus Canopy Persists 38 Yr in an A Scophyllum‐Dominated Community.” Ecosphere 8: e01725.

[ece370491-bib-0059] Menge, B. A. , F. Chan , S. Dudas , et al. 2009a. “Do Terrestrial Ecologists Ignore Aquatic Literature?” Frontiers in Ecology and the Environment 7: 182–183.

[ece370491-bib-0060] Menge, B. A. , F. Chan , S. Dudas , et al. 2009b. “Terrestrial Ecologists Ignore Aquatic Literature: Asymmetry in Citation Breadth in Ecological Publications and Implications for Generality and Progress in Ecology.” Journal of Experimental Marine Biology and Ecology 377: 93–100.

[ece370491-bib-0061] Miller, E. J. 2015. “Ecology of Hector's Dolphin (*Cephalorhynchus hectori*): Quantifying Diet and Investigating Habitat Selection at Banks Peninsula.”

[ece370491-bib-0062] Mittelbach, G. G. , and B. J. McGill . 2019. Community Ecology. New York: Oxford University Press.

[ece370491-bib-0063] Molles, M. C., Jr. , and A. A. Sher . 2019. Ecology: Concepts and Applications. 8th ed. New York: McGraw‐Hill Education.

[ece370491-bib-0064] Murray, S. N. , and T. G. Denis . 1997. “Vulnerability, of the Rockweed *Pelvetia compressa* to Anthropogenic Disturbance on Southern California Rocky Shores.” Phycologia 36: 75–76.

[ece370491-bib-0065] Pessarrodona, A. 2022. “Functional Extinction of a Genus of Canopy‐Forming Macroalgae (*Cystophora* spp.) Across Western Australia.” Regional Environmental Change 22: 130.

[ece370491-bib-0066] Pessarrodona, A. , J. Assis , K. Filbee‐Dexter , et al. 2022. “Global Seaweed Productivity.” Science Advances 8: eabn2465. 36103524 10.1126/sciadv.abn2465 PMC9473579

[ece370491-bib-0067] Pessarrodona, A. , and C. M. Grimaldi . 2022. “On the Ecology of *Cystophora* spp. Forests.” Journal of Phycology 58: 760–772. 36054376 10.1111/jpy.13285 PMC10092567

[ece370491-bib-0068] Ray, G. C. , and J. McCormick‐Ray . 2013. Marine Conservation: Science, Policy, and Management. Hoboken, NJ: John Wiley & Sons.

[ece370491-bib-0069] Rendina, F. , A. Falace , G. Alongi , et al. 2023. “The Lush Fucales Underwater Forests off the Cilento Coast: An Overlooked Mediterranean Biodiversity Hotspot.” Plants 12: 1497. 37050123 10.3390/plants12071497 PMC10096796

[ece370491-bib-0070] Schagerström, E. , H. Forslund , L. Kautsky , M. Pärnoja , and J. Kotta . 2014. “Does Thalli Complexity and Biomass Affect the Associated Flora and Fauna of Two Co‐Occurring *Fucus* Species in the Baltic Sea?” Estuarine, Coastal and Shelf Science 149: 187–193.

[ece370491-bib-0071] Schiel, D. R. 1990. “Macroalgal Assemblages in New Zealand: Structure, Interactions and Demography.” Hydrobiologia 192: 59–76.

[ece370491-bib-0072] Schiel, D. R. , T. Alestra , S. Gerrity , et al. 2019. “The Kaikōura Earthquake in Southern New Zealand: Loss of Connectivity of Marine Communities and the Necessity of a Cross‐Ecosystem Perspective.” Aquatic Conservation: Marine and Freshwater Ecosystems 29: 1520–1534.

[ece370491-bib-0073] Schiel, D. R. , and M. S. Foster . 2006. “The Population Biology of Large Brown Seaweeds: Ecological Consequences of Multiphase Life Histories in Dynamic Coastal Environments.” Annual Review of Ecology, Evolution, and Systematics 37: 343–372.

[ece370491-bib-0074] Smale, D. , M. S. Thomsen , S. Bennett , et al. 2023. “Biodiversity and Ecosystem Services.” United Nations Environment Programme. Into the Blue: Securing a Sustainable Future for Kelp Forests. Nairobi:45‐70.

[ece370491-bib-0075] Smale, D. A. , M. T. Burrows , P. Moore , N. O'Connor , and S. J. Hawkins . 2013. “Threats and Knowledge Gaps for Ecosystem Services Provided by Kelp Forests: A Northeast A Tlantic Perspective.” Ecology and Evolution 3: 4016–4038. 24198956 10.1002/ece3.774 PMC3810891

[ece370491-bib-0076] Smale, D. A. , and T. Wernberg . 2013. “Extreme Climatic Event Drives Range Contraction of a Habitat‐Forming Species.” Proceedings of the Royal Society B: Biological Sciences 280: 20122829. 10.1098/rspb.2012.2829 PMC3574333 23325774

[ece370491-bib-0077] Smith, T. M. , and R. L. Smith . 2015. Elements of Ecology. 9th ed. Harlow, Essex: Pearson Education Limited.

[ece370491-bib-0078] Steinberg, P. D. , J. A. Estes , and F. C. Winter . 1995. “Evolutionary Consequences of Food Chain Length in Kelp Forest Communities.” Proceedings of the National Academy of Sciences 92: 8145–8148. 10.1073/pnas.92.18.8145 PMC41112 11607573

[ece370491-bib-0079] Tegner, M. , and P. Dayton . 2000. “Ecosystem Effects of Fishing in Kelp Forest Communities.” ICES Journal of Marine Science 57: 579–589.

[ece370491-bib-0080] Thomsen, M. S. , A. H. Altieri , C. Angelini , et al. 2022. “Heterogeneity Within and Among Co‐Occurring Foundation Species Increases Biodiversity.” Nature Communications 13: 1–9. 10.1038/s41467-022-28194-y PMC8803935 35102155

[ece370491-bib-0081] Thomsen, M. S. , I. Metcalfe , P. South , and D. R. Schiel . 2015. “A Host‐Specific Habitat Former Controls Biodiversity Across Ecological Transitions in a Rocky Intertidal Facilitation Cascade.” Marine and Freshwater Research 67: 144–152.

[ece370491-bib-0082] Thomsen, M. S. , L. Mondardini , F. Thoral , et al. 2021. “Cascading Impacts of Earthquakes and Extreme Heatwaves Have Destroyed Populations of an Iconic Marine Foundation Species.” Diversity and Distributions 27: 2369–2383.

[ece370491-bib-0083] Thomsen, M. S. , and P. M. South . 2019. “Communities and Attachment Networks Associated With Primary, Secondary and Alternative Foundation Species: A Case Study of Stressed and Disturbed Stands of Southern Bull Kelp.” Diversity 11: 56.

[ece370491-bib-0084] Tokeshi, M. , and S. Arakaki . 2012. “Habitat Complexity in Aquatic Systems: Fractals and Beyond.” Hydrobiologia 685: 27–47.

[ece370491-bib-0085] Torn, K. , D. Krause‐Jensen , and G. Martin . 2006. “Present and Past Depth Distribution of Bladderwrack (*Fucus vesiculosus*) in the Baltic Sea.” Aquatic Botany 84: 53–62.

[ece370491-bib-0086] Tuya, F. , and R. J. Haroun . 2006. “Spatial Patterns and Response to Wave Exposure of Shallow Water Algal Assemblages Across the Canarian Archipelago: A Multi‐Scaled Approach.” Marine Ecology Progress Series 311: 15–28.

[ece370491-bib-0087] Ugarte, R. A. , and G. Sharp . 2001. “6. A New Approach to Seaweed Management in Eastern Canada: The Case of *Ascophyllum nodosum* .” Cahiers de Biologie Marine 42: 63–70.

[ece370491-bib-0088] Valdazo, J. , M. A. Viera‐Rodríguez , F. Espino , R. Haroun , and F. Tuya . 2017. “Massive Decline of *Cystoseira abies*‐Marina Forests in Gran Canaria Island (Canary Islands, Eastern Atlantic).” Scientia Marina 81: 499–507.

[ece370491-bib-0089] Valdazo, J. , M. A. Viera‐Rodríguez , and F. Tuya . 2020. “Seasonality in the Canopy Structure of the Endangered Brown Macroalga *Cystoseira abies*‐Marina at Gran Canaria Island (Canary Islands, Eastern Atlantic).” European Journal of Phycology 55: 253–265.

[ece370491-bib-0090] Van Alstyne, K. , J. McCarthy III , C. Hustead , and D. Duggins . 1999a. “Geographic Variation in Polyphenolic Levels of Northeastern Pacific Kelps and Rockweeds.” Marine Biology 133: 371–379.

[ece370491-bib-0091] Van Alstyne, K. L. , J. J. McCarthy III , C. L. Hustead , and L. J. Kearns . 1999b. “Phlorotannin Allocation Among Tissues of Northeastern Pacific Kelps and Rockweeds.” Journal of Phycology 35: 483–492.

[ece370491-bib-0092] Vasconcelos, J. B. , T. N. de Vasconcelos Reis , A. de Lourdes Montenegro Cocentino , et al. 2019. “Macroalgal Responses to Coastal Urbanization: Relative Abundance of Indicator Species.” Journal of Applied Phycology 31: 893–903.

[ece370491-bib-0093] Vaux, F. , C. I. Fraser , D. Craw , S. Read , and J. M. Waters . 2023. “Integrating Kelp Genomic Analyses and Geological Data to Reveal Ancient Earthquake Impacts.” Journal of the Royal Society Interface 20: 20230105. 37194268 10.1098/rsif.2023.0105 PMC10189309

[ece370491-bib-0094] Verdura, J. , J. Santamaría , E. Ballesteros , et al. 2021. “Local‐Scale Climatic Refugia Offer Sanctuary for a Habitat‐Forming Species During a Marine Heatwave.” Journal of Ecology 109: 1758–1773.

[ece370491-bib-0095] Vergés, A. , A. H. Campbell , G. Wood , et al. 2020. “Operation Crayweed: Ecological and Sociocultural Aspects of Restoring Sydney's Underwater Forests.” Ecological Management & Restoration 21: 74–85.

[ece370491-bib-0096] Verhoef, H. A. , and P. J. Morin . 2010. Community Ecology: Processes, Models, and Applications. New York: Oxford University Press.

[ece370491-bib-0097] Wang, M. , C. Hu , B. B. Barnes , G. Mitchum , B. Lapointe , and J. P. Montoya . 2019. “The Great Atlantic *Sargassum* Belt.” Science 365: 83–87. 31273122 10.1126/science.aaw7912

[ece370491-bib-0098] Warham, J. 1974. “The Breeding Biology and Behaviour of the Snares Crested Penguin.” Journal of the Royal Society of New Zealand 4: 63–108.

[ece370491-bib-0099] Webb, T. J. 2012. “Marine and Terrestrial Ecology: Unifying Concepts, Revealing Differences.” Trends in Ecology & Evolution 27: 535–541. 22795608 10.1016/j.tree.2012.06.002

[ece370491-bib-0100] Wernberg, T. , and K. Filbee‐Dexter . 2019. “Missing the Marine Forest for the Trees.” Marine Ecology Progress Series 612: 209–215.

[ece370491-bib-0101] Wernberg, T. , M. S. Thomsen , J. Baum , et al. 2024. “Impacts of Climate Change on Marine Foundation Species.” Annual Review of Marine Science 16: 247–282. 10.1146/annurev-marine-042023-093037 37683273

[ece370491-bib-0102] Whitaker, S. G. , R. F. Ambrose , L. M. Anderson , et al. 2023. “Ecological Restoration Using Intertidal Foundation Species: Considerations and Potential for Rockweed Restoration.” Ecosphere 14: e4411.

[ece370491-bib-0103] Witherington, B. , S. Hirama , and R. Hardy . 2012. “Young Sea Turtles of the Pelagic *Sargassum*‐Dominated Drift Community: Habitat Use, Population Density, and Threats.” Marine Ecology Progress Series 463: 1–22.

